# Population-based input function (PBIF) applied to dynamic whole-body 68Ga-DOTATOC-PET/CT acquisition

**DOI:** 10.3389/fnume.2022.941848

**Published:** 2022-09-21

**Authors:** Philippe Thuillier, David Bourhis, Mathieu Pavoine, Jean-Philippe Metges, Romain Le Pennec, Ulrike Schick, Frédérique Blanc-Béguin, Simon Hennebicq, Pierre-Yves Salaun, Véronique Kerlan, Nicolas A. Karakatsanis, Ronan Abgral

**Affiliations:** ^1^ Department of Endocrinology, University Hospital of Brest, Brest, France; ^2^ UMR 1304 Inserm GETBO, University Hospital of Brest, Brest, France; ^3^ Department of Nuclear Medicine, University Hospital of Brest, Brest, France; ^4^ Department of Oncology, University Hospital of Brest, Brest, France; ^5^ Department of Radiotherapy, University Hospital of Brest, Brest, France; ^6^ Department of Radiology, Weil Cornell Medical College of Cornell University, New York, NY, United States

**Keywords:** neuroendocrine tumor, dynamic whole-body acquisition, 68Ga-DOTATOC-PET/CT, population-based input function, net influx rate

## Abstract

**Rational:**

To validate a population-based input function (PBIF) model that alleviates the need for scanning since injection time in dynamic whole-body (WBdyn) PET.

**Methods:**

Thirty-seven patients with suspected/known well-differentiated neuroendocrine tumors were included (GAPETNET trial NTC03576040). All WBdyn 68Ga-DOTATOC-PET/CT acquisitions were performed on a digital PET system (one heart-centered 6 min-step followed by nine WB-passes). The PBIF model was built from 20 image-derived input functions (IDIFs) obtained from a respective number of patients’ WBdyn exams using an automated left-ventricle segmentation tool. All IDIF peaks were aligned to the median time-to-peak, normalized to patient weight and administrated activity, and then fitted to an exponential model function. PBIF was then applied to 17 independent patient studies by scaling it to match the respective IDIF section at 20–55 min post-injection time windows corresponding to WB-passes 3–7. The ratio of area under the curves (AUCs) of IDIFs and PBIF_3–7_ were compared using a Bland–Altman analysis (mean bias ± SD). The Patlak-estimated mean Ki for physiological uptake (Ki-liver and Ki-spleen) and tumor lesions (Ki-tumor) using either IDIF or PBIF were also compared.

**Results:**

The mean AUC ratio (PBIF/IDIF) was 0.98 ± 0.06. The mean Ki bias between PBIF_3–7_ and IDIF was −2.6 ± 6.2% (confidence interval, CI: −5.8; 0.6). For Ki-spleen and Ki-tumor, low relative bias with low SD were found [4.65 ± 7.59% (CI: 0.26; 9.03) and 3.70 ± 8.29% (CI: −1.09; 8.49) respectively]. For Ki-liver analysis, relative bias and SD were slightly higher [7.43 ± 13.13% (CI: −0.15; 15.01)].

**Conclusion:**

Our study showed that the PBIF approach allows for reduction in WBdyn DOTATOC-PET/CT acquisition times with a minimum gain of 20 min.

## Introduction

Neuroendocrine tumors (NETs) are a group of rare tumors with a common embryological origin (1). NETs are characterized by cellular overexpression of somatostatin receptors (SSTr) allowing the use of radio-labeled somatostatin analogs for diagnostic imaging or peptide-receptor radionuclide therapy. Currently, Gallium-68 DOTA-conjugated somatostatin receptor-targeted peptide (68Ga-DOTA-SSTr) positron emission tomography computed tomography (PET/CT) is the mainstay of well-differentiated NET (WD-NET) diagnosis, staging, and monitoring (2, 3). Dynamic whole-body (WBdyn) acquisition methods in PET imaging have been proposed to assess the spatiotemporal distribution of radiotracers across the human body, allowing calculation of kinetic parameters of clinical relevance, such as the tracer uptake rate Ki using Patlak analysis (4–6). In a recent prospective study, our team showed the feasibility of a WBdyn acquisition in 68Ga-DOTATOC-PET/CT in 61 patients (7). The realization of Ki parametric images required a long dynamic PET acquisition (i.e., 45–60 min depending on the radiotracer) with tracer injection under the PET system to obtain the blood input function and to model time–activity curves in physiological and tumor tissues (8, 9). Currently, several solutions have been developed to make the procedure less constraining such as image-derived input function (IDIF) estimation that is less invasive than venous blood sampling (10, 11). Nevertheless, the main issue in using the Patlak model remains the need to know the time integral of the radiotracer's plasma concentration since injection time throughout the images acquisition (12). One of the current challenges is to reduce this long acquisition time to optimize WBdyn PET studies. Several recent WBdyn 18F-FDG-PET/CT studies have proposed the use of a population-based input function (PBIF) to overcome this key issue (13–19), but to our knowledge, no such data with 68Ga-DOTA-SSTr tracers are available.

The aims of this study were to develop a PBIF model in WBdyn 68Ga-DOTATOC-PET/CT and to validate its clinical use by the most accurate possible estimation of Ki parametric images from fewer WB-passes in a WB-NET independent cohort.

## Methods

### Patient population

This is an ancillary study of GAPETNET trial (NTC03576040), which is a prospective, observational, and single-center cohort study.

Inclusion criteria were as follows: age ≥18 years old; well-differentiated grade 1 or 2 (G1 or G2) neuroendocrine tumor; primary location: gastroenteropancreatic, bronchopulmonary, or unknown; and WBdyn 68Ga-DOTATOC-PET/CT acquisition performed on digital PET. The protocol was approved by the Institutional Medical Ethics Committee of Brest (29BRC17.0036). Informed consent was obtained from all the patients to participate in the study.

A total of 37 subjects were recruited for this study. The subjects were divided into two groups: a PBIF modeling group (*n* = 20) and an independent validation cohort (*n* = 17).

### PET/CT acquisition and image reconstruction

All WBdyn 68Ga-DOTATOC-PET/CT acquisitions were performed on two digital Biograph Vision 600 systems (Siemens©, Erlangen, Germany).

CT acquisition was performed after injection of intravenous iodine contrast agent (1.5 ml/kg), unless contraindicated. The CT consisted of a 64-slice multidetector-row spiral scanner with a transverse field of view of 500 mm. The CT images were reconstructed with an iterative method (SAFIRE, strength 5) for image interpretation. An additional reconstruction of the CT data was performed for attenuation correction using a filtered back projection algorithm and a 780 mm diameter to avoid truncation artifacts.

#### Dynamic whole-body PET protocol

PET images were then acquired immediately after a manual injection of 68Ga-DOTATOC. The WBdyn PET acquisition was performed according to the methodology previously described by Karakatsanis et al. (5, 6, 9, 20).

A single dynamic cardiac-bed (DCB) position acquisition was followed by a WBdyn craniocaudal continuous bed motion acquisition: 6-min DCB (12 images × 5 s, 6 images × 10 s, 8 images × 30 s) + WBdyn acquisition (9 passes with 2.2 mm/s ≈ 54-min duration for the whole WBdyn acquisition).

PET data were first reconstructed with attenuation correction using an iterative reconstruction algorithm (OSEM 3D) with time of flight (ToF) and point spread function (PSF) correction (TrueX). PET images were corrected for random coincidence, scatter, deadtime, normalization, isotope decay, and attenuation using CT data; no smoothing was applied post reconstruction. The size of the transaxial reconstruction was 440 × 440 (voxel size = 1.65 mm × 1.65 mm × 1.65 mm) with three iterations, five subsets, and 2 mm Gaussian post-filtering. Second, dynamic data were reconstructed using 4D nested direct Patlak reconstruction, matrix 220 × 220, four iterations, five subsets, and 3 mm Gaussian post-filtering, thanks to the IDIF or PBIF described below.

#### Input function

Theoretically, an arterial blood sample is required to obtain an IF, but several studies have shown that it can be estimated only from image data (21–25).

As previously described (26), the total radioactivity concentration in the whole blood is quantified in the reconstructed image and used for the extraction of the IDIF and later for building the PBIF model. A sphere of radius 12 mm was automatically generated as close as possible at the center of the left ventricle and away from the myocardium to mitigate any partial volume effects (26) on both CT series corresponding to DCB and WBdyn acquisitions using an ALPHA [Automated Learning and Parsing of Human Anatomy (27)] algorithm and then applied on PET reconstructed images.

Because we did not performed arterial or venous blood tests during image acquisition, we decided to apply a fixed plasma-to-whole-blood ratio to correct for the IDIF by multiplying by 1.6 the arterial time–activity concentration curve, as previously reported (24, 28).

### PBIF creation and IDIF validation cohort

#### PBIF modeling and fitting (group 1)

The first step was to model a PBIF from the IDIFs of the 20 patients in group 1 as follows: first, all IDIF peaks were aligned to the median time-to-peak (TTP); then, all IDIFs were normalized to the patient’s weight and administered activity; finally, the mean IDIF value at each time was used to model the PBIF (Supplementary Figure S1).

The second step was to fit the PBIF using a mathematical model. For this, the PBIF was fitted with a linear interpolation of the concentration before TTP and with an exponential model function using Labfit software® after TTP as follows:

$$SUV\left(t\right)=\left\{ \begin{array}{cc} 0.54\left(t\right)-5.54 & \mathrm{If}\;t<\mathrm{TTP},\\ e^{2.27+\frac{22.33}{t}-0.47\ln(t)} & \mathrm{If}\;t\;\geq \mathrm{TTP} \end{array}\right.$$

where *t* is in seconds.

#### Validation cohort (group 2)

The third step was to use the modeling PBIF in group 2, including 17 independent patients. The modeled PBIF had to be scaled for each patient and a scaling factor was calculated using the tail part of the IDIF, from the third to seventh passes. For each patient, the PBIF was scaled so that the area under the curve matched the IDIF from the third to seventh WB-passes (PBIF_3–7_) corresponding approximately to 20–55 min time windows. We used PBIF_3–7_ because Patlak plots were also calculated from the third to seventh passes of the WBdyn acquisition (7).

An example of the process is illustrated in Supplementary Figure S2.

### Patlak reconstruction and Ki extraction

Patlak reconstructions were performed using 4D nested generalized Patlak expectation-maximization reconstruction in the validation cohort, using both IDIF and the scaled PBIFs to obtain parametric images (5, 29–31). Mean Ki-Liver, Ki-spleen, and Ki-tumor values (in ml/min/100 ml) were generated from the different reconstructed parametric images by applying circular 3- and 1-cm diameter region of interests (ROI), respectively, drawn over an uninvaded part of the liver (in the right hepatic lobe) and spleen organs, as previously recommended (32); spherical volume of interests (VOI) drawn over the highest tumor uptake using a fixed threshold method delineating a 3D contour around voxels equal to or greater than 40% of the lesion Ki max, by analogy with the SUV approach. Each ROI and VOI were segmented on the last ninth frame and applied to generate time–activity curves.

**Figure 1:**
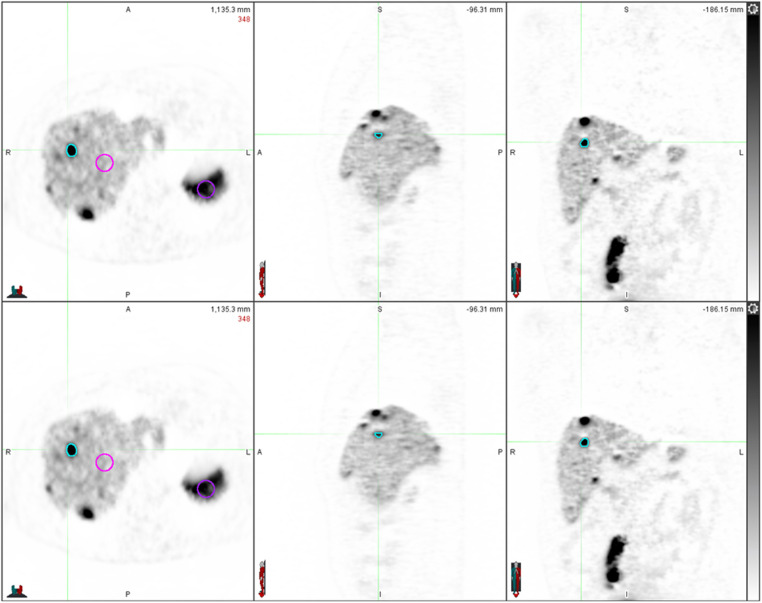
Parametric images reconstructed with IDIF on top and with PBIF3-7 on the bottom.

An example of parametric images reconstructed using both IDIF and PBIF is represented in the Figure 1.

### Statistical analysis

The performance of each scaled PBIF was compared one-by-one to the corresponding IDIF with AUCs. The mean Ki-Liver, Ki-spleen, and Ki-tumor values using both PBIF and IDIF were also compared together. AUC ratio (AUC_PBIF/IDIF_) and Ki ratio (Ki_PBIF/IDIF_) were calculated. Data comparisons were performed using a linear correlation test (*R*^2^ − slope) and a Bland–Altman analysis [mean relative bias; confidence interval (CI) 95%].

The significance level of the *p*-value was 0.05. All statistical analyses were performed using XLStat 2021 (Addinsoft©, Paris, France) and Excel (Microsoft©, Redmond, Washington, United States) software.

## Results

### Population

The mean injected tracer doses were 204 ± 45 MBq (range: 136–295) and 2.67 ± 0.29 MBq/Kg (range: 2.02–3.06) in the PBIF modeling group. The mean injected tracer doses were 199 ± 46 MBq (range: 108–287) and 2.81 ± 0.28 MBq/Kg (range: 2.08–3.29) in the validation cohort. There were no significant differences in the demographics parameters (age, sex ratio, and body mass index) between the PBIF modeling group and the independent validation cohort.

### WBdyn PET results

#### Scaled PBIFs with IDIF comparison

The mean AUC_PBIF/IDIF_ ratio was 0.98 ± 0.06 (range: 0.88–1.11) and *R*^2^ = 0.96 (slope = 1.00).

**Figure 2:**
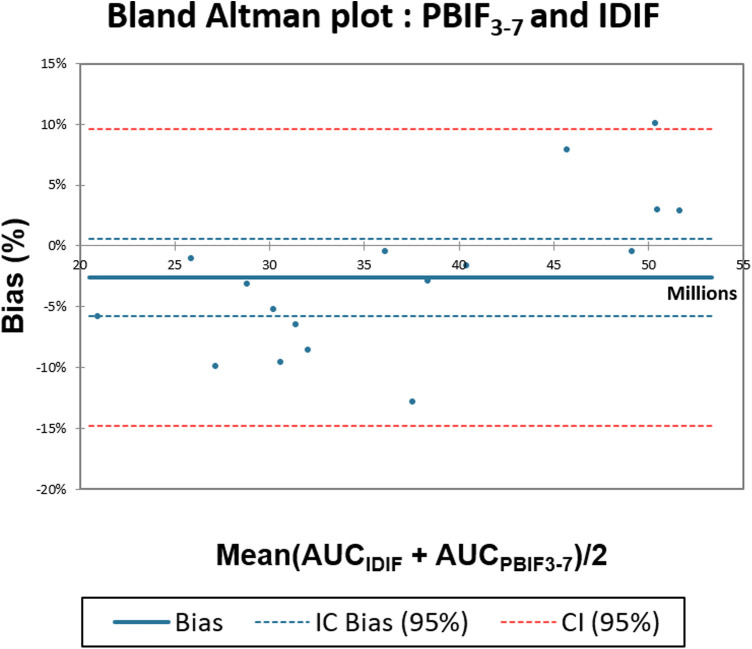
Comparison of AUC_IDIF_ with AUC_PBIF3-7_ using IDIF Bland–Altman plot.

The mean relative bias ± SD between PBIF_3–7_ and IDIF was −2.6 ± 6.2% (CI: −5.8; 0.6) (Figure 2).

#### Ki values analysis

The mean Ki_PBIF/IDIF_ ratio, *R*^2^ correlation coefficient, and mean relative bias ± SD between PBIF and IDIF are presented in Table 1.

**Table 1:** Mean Ki ratio of Ki-liver, Ki-spleen, and Ki-tumor with results of the correlation test and Bland–Altman analysis between PBIF_3−7_ and IDIF.

	Mean Ki (PBIF/IDIF) [mean ± SD (range)]	Correlation analysis *R*^2^ (slope)	Mean relative bias [mean ± SD (CI)]
Ki-liver	1.09 ± 0.14 (0.80–1.31)	0.80 (1.08)	7.43 ± 13.13% (−0.15; 15.01)
Ki-spleen	1.05 ± 0.08 (0.91–1.18)	0.96 (1.05)	4.65 ± 7.59% (0.26; 9.03)
Ki-tumor	1.04 ± 0.09 (0.92–1.26)	1.00 (1.02)	3.70 ± 8.29% (−1.09; 8.49)

PBIF, population-based input function; IDIF, image-derived input function; CI, confidence interval.

**Figure 3:**
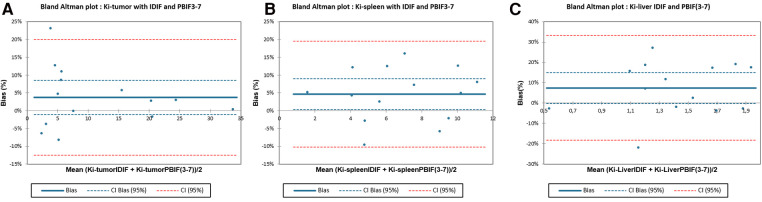
Comparison between Ki-liver, Ki-spleen, and Ki-tumor calculated with IDIF versus PBIF3-7 using Bland–Altman plot.

The lowest relative bias ± SD were found for Ki-tumor [3.70 ± 8.29% (CI: −1.09; 8.49)] and Ki-spleen [4.65 ± 7.59% (CI: 0.26; 9.03)] metrics comparison (Figures 3A,B). For Ki-liver analysis, the relative bias and SD were slightly higher [7.43 ± 13.13% (CI: −0.15; 15.01)] (Figure 3C).

## Discussion

In our study, we showed the feasibility of using a PBIF to estimate Ki values of WD-NETs from fewer whole-body passes in WBdyn 68Ga-DOTATOC-PET/CT. To our knowledge, this is the first study with such a 68Ga-DOTA-SSTr tracer comparing the PBIF and IDIF approaches for Patlak analysis in WBdyn PET/CT. Implementation of routine WBdyn acquisition for patients with WD-NETs could lead to a better characterization of the physiological and tumoral tracer uptake in the future, providing *in vivo* additional quantitative value in the characterization of SSTR expression in WD-NETs as previously reported (7).

Regarding the comparison between IDIF and scaled PBIF_3–7_, we found a low bias [AUC ratio = 0.98 ± 0.06, *R*^2^ = 0.96 (slope = 1.00), mean bias −2.6 ± 6.2%]. In comparison with literature data, Naganawa et al. compared IDIF and PBIF in oncological WBdyn 18F-FDG-PET/CT studies with the gold standard as an arterial input function (AIF). The authors highlighted that using the PBIF on 15–45 and 30–60 min time windows allowed us to obtained respective mean bias of −1 ± 6% (*R*^2^ = 0.93) and 3 ± 6% (*R*^2^ = 0.94) compared to AIF (13). These time windows are finally quite like the one used in our study (20–55 min). Moreover, they found similar results in using the IDIF with a bias of −1 ± 5% (*R*^2^ = 0.91). However, the choice of later time windows (45–75 and 60–90 min) led to an overestimation of the AIF in their series [mean bias of 9 ± 7%, (*R*^2^ = 0.93) and 19 ± 10% (*R*^2^ = 0.88), respectively]. Therefore, we can assume that in terms of AUC ratio, using a 3–7 time window does not lead to a significant overall impact compared to IDIF.

Our results showed excellent results for Ki-tumor estimation with PBIF providing low bias with low standard deviation. Moreover, the confidence interval of this relative bias included the value 0 in our comparison [bias = 3.70 ± 8.29% (CI: −1.09; 8.49)] that is of major importance in a context of optimizing the characterization of NETs in 68Ga-DOTATOC-PET/CT. In a recent study including 14 patients with lung cancer, Indovina et al. found similar results on Ki-metrics estimation using a PBIF model scaled on a 40–60 min time window in WBdyn 18F-FDG-PET/CT (correlation of mean Ki_IDIF_ and Ki_PBIF_ values of *R*^2^ = 0.997) (14).

We also studied physiological Ki-metrics because spleen and liver uptakes were historically used in scintigraphy to estimate SSTr density expression in NETs (33). Using PBIF_3–7_, we found relatively same results for Ki-spleen than for Ki-tumor estimation with a low relative bias [Ki ratio = 1.05 ± 0.08, *R*^2^ = 0.96 (slope = 1.05), bias = 4.66 ± 7.59% (CI: 0.26; 9.03)]. However, for Ki-liver analysis, we found a positive bias with higher SD of 7.43 ± 13.13%. In our cohort, mean Ki-liver values were lower to the mean Ki-spleen and Ki-tumor values in our 17 scans (1.35 ± 0.36, 6.65 ± 2.77, and 11.09 ± 9.87 ml/min/100 ml, respectively). So, we assume that higher variance of bias in our Ki-liver analysis is mainly related to their low values, whose small changes had a greater impact in modeling the input function. Finally, it might also be explained by physiological motion of such organ (i.e., breathing) during data acquisition.

Our study has several limitations. First, we did not perform arterial blood sample for PBIF scaling. Hence, our analysis allows only a comparison to the IDIF-based approach. Therefore, we cannot conclude whether the use of PBIF results in more accurate estimation of Ki values compared to the AIF gold standard. However, we applied a fixed plasma-to-whole-blood ratio of 1.6 to correct for the IDIF as previously reported, which leads to estimate accurately the Ki values without performing iterative arterial or venous blood tests during image acquisition (24). Furthermore, we only used the WB-passes time window proposed by our reconstruction software. The comparison of results using different time windows will be the topic of a future publication to find out what is the best compromise between the acquisition time duration and the most optimized PBIF in terms of bias and variance. Finally, due to the relatively small number of patients, we did not perform subgroup analysis such as with patient age- or gender-specific IFs that could improve the accuracy of the PBIF modeling.

## Conclusion

Our study showed that the PBIF approach allows for a reduction in WBdyn DOTATOC-PET/CT acquisition time, allowing a minimum time gain of 20 min using WB-passes 3–7 and thereby facilitating its use in routine clinical practice. Further evaluation with a larger dataset is needed to confirm these promising results.

## Data Availability

The raw data supporting the conclusions of this article will be made available by the authors, without undue reservation.

## Supplementary material

Supplementary Figure 1 Mean ± SD of the 20 IDIFs of the group 1 and corresponding modelized PBIF.

Supplementary Figure 2 Example of nonscaled PBIF and IDIF for the full acquisition (top) and the scaled PBIF using 3–7 pass points from the IDIF (bottom).

Supplementary Table 1 General characteristics of the PBIF creation (group 1) and IDIF validation population (group 2).
