# Supplementary figures and images for: Population-based input function (PBIF) applied to dynamic whole-body 68Ga-DOTATOC-PET/CT acquisition
Source: Front Nucl Med. 2022 Sep 21;2:941848. doi: 10.3389/fnume.2022.941848 (PMC11464975; doi:10.3389/fnume.2022.941848)

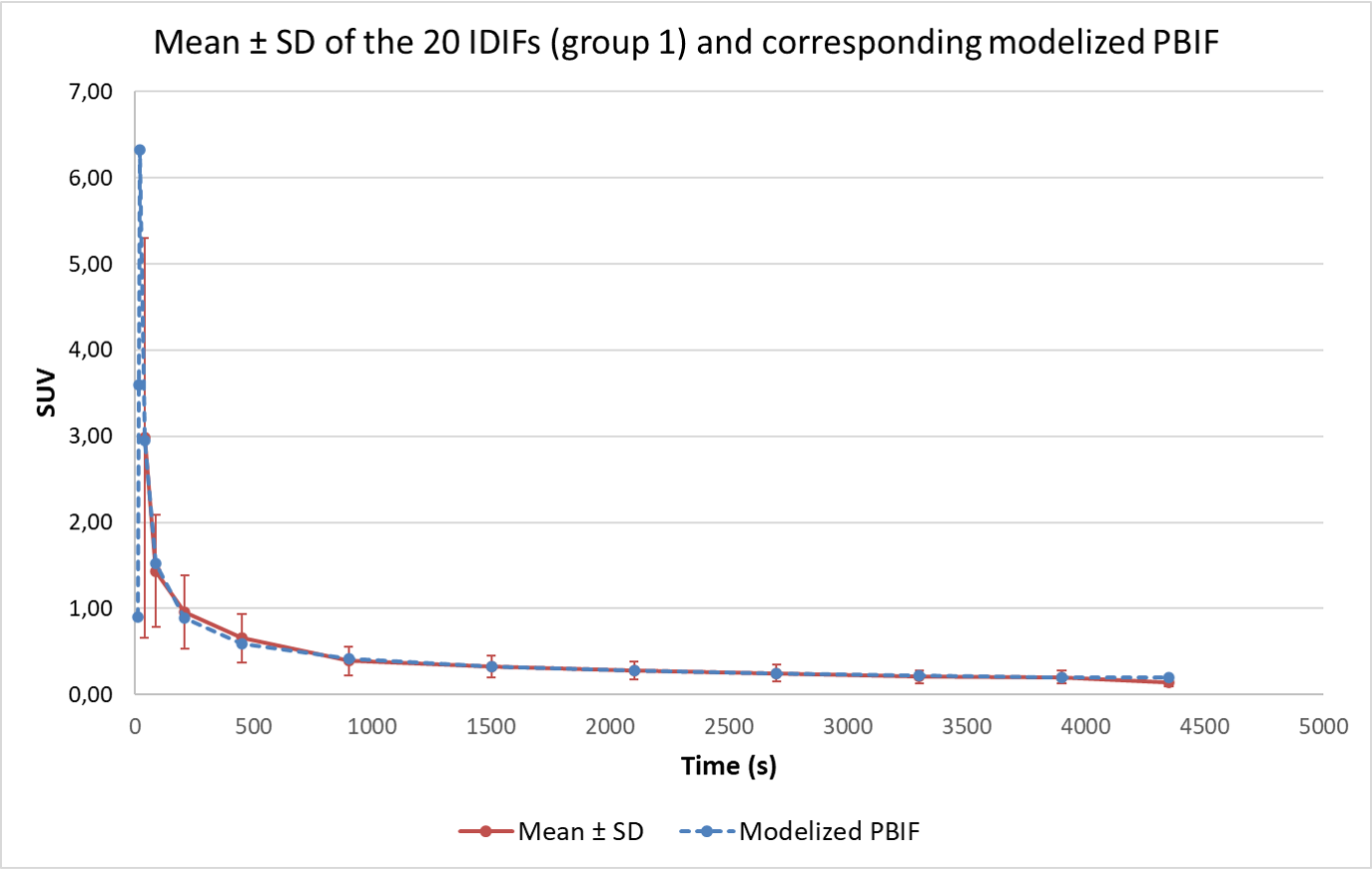

Supplement: Supplementary Figure 1 — Mean ± SD of the 20 IDIFs of the group 1 and corresponding modelized PBIF. [file Image1.tif]

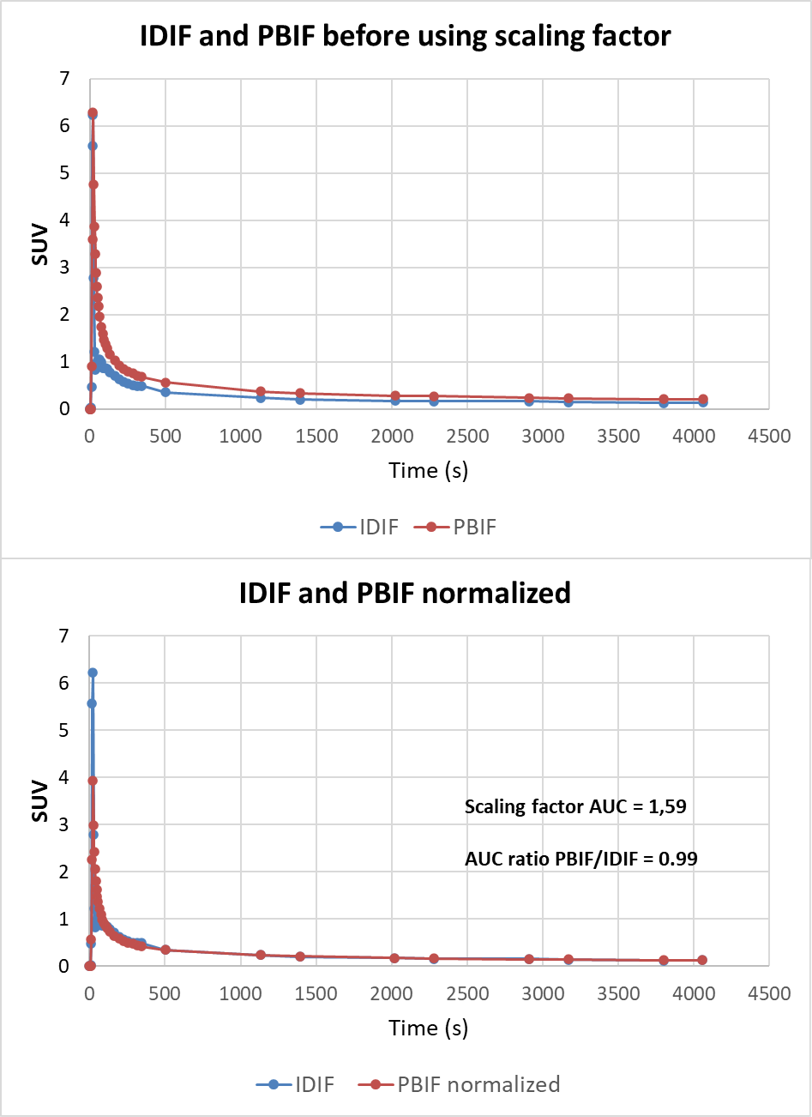

Supplement: Supplementary Figure 2 — Example of nonscaled PBIF and IDIF for the full acquisition (top) and the scaled PBIF using 3–7 pass points from the IDIF (bottom). [file Image2.tif]
