# Supplementary material for: Population-based input function (PBIF) applied to dynamic whole-body 68Ga-DOTATOC-PET/CT acquisition
Source: Front Nucl Med. 2022 Sep 21;2:941848. doi: 10.3389/fnume.2022.941848 (PMC11464975; doi:10.3389/fnume.2022.941848)
Supplement: Supplementary Table 1 — General characteristics of the PBIF creation (group 1) and IDIF validation population (group 2). [file Table1.docx]

| Characteristics | PBIF modeling group (n=20) | Validation cohort (n=17) | pvalue |
| --- | --- | --- | --- |
| Age (yo; mean ± SD) | 63.55±15.58 | 67.06 ±9.3 | 0.422 |
| Gender (%M/%F) | 12(60%)/8(40%) | 8(47.06%)/9(52.94%) | 0.431 |
| Primary tumor |  |  | 0.252 |
| Pancreas | 9 (45%) | 4 (23.53%) |  |
| Digestive | 10 (50%) | 9 (52.94%) |  |
| Lung | 0 (0%) | 2 (11.77%) |  |
| Other/unknown | 1 (5%) | 2 (11.77%) |  |
| Tumor grade* |  |  | 0.153 |
| G1 | 6 (35.3) | 8 (61.5) |  |
| G2 | 11 (64.7) | 5 (38.5) |  |
| Height**(cm; mean ± SD) | 168.53 ± 69.12 | 168.92±96.4 | 0.910 |
| Weight (Kg; mean ± SD) | 75.5±17.86 | 71.30±16.74 | 0.467 |
| Body mass Index  (Kg/m²; mean ± SD) | 26.47± 5.89 | 24.38 ± 4.92 | 0.322 |
| Activity injected  (Mbeq; mean ± SD)) | 199.15 ± 44.92 | 199.29 ±46.34 | 0.992 |
| Activity injected/kg  (Mbeq/kg; mean ± SD)) | 2.66 ± 0.29 | 2.81 ± 0.28 | 0.137 |

*missing data (n=5 in group1 and n=4 in group 2)
**missing data (n=3 in group1 and n=4 in group 2)

Supplemental table 1: General characteristics of the PBIF creation (group 1) and IDIF validation population (group 2)
